# Physiological evaluation of the behavior and epidermis of freshwater planarians (*Girardia tigrina* and *Girardia* sp.) exposed to stressors

**DOI:** 10.1242/bio.029595

**Published:** 2018-06-15

**Authors:** Matheus Salgado de Oliveira, Karla Andressa Ruiz Lopes, Priscila Maria Sarmeiro Corrêa Marciano Leite, Flavia Villaça Morais, Nádia Maria Rodrigues de Campos Velho

**Affiliations:** 1 Planarian Laboratory, Nature Research Center, Faculty of Education and Arts, University of Vale do Paraíba, São José dos Campos, São Paulo 12244-000, Brazil; 2 Research and Development Institute, University of Vale do Paraíba, São José dos Campos, São Paulo 12244-000, Brazil; 3 Laboratory of Cellular and Molecular Biology of Fungi, Research and Development Institute, University of Vale do Paraíba, São José dos Campos, São Paulo 12244-000, Brazil

**Keywords:** Platyhelminthes, Physiology, Epidermis, Stressors

## Abstract

Planarians are metazoan freshwater flatworms which are free-living organisms. Their body has pluripotent stem cell promoters of tissue regeneration capacity. The water temperature and the potential of hydrogen (pH) of lentic ecosystems are important factors involved in the distribution and abundance of these animals. Although the pH factor is directly related to the physiology and behavior of planarians, their adaptive and regenerating capacities still remain unknown. The Critical Thermal Maximum (CTM) is a very widespread method used in the evaluation of thermal tolerance. In this study, *Girardia tigrina* (Girard, 1850) and *Girardia* sp., a species found in Brazil, which is under study as a new species, had their epidermis assessed by scanning electron microscopy (SEM) to analyze their physiological structures before and after exposure to different stressors. SEM was used as a method to evaluate the planarians' epidermis as a result of the increasing temperature (CTM) and pH alterations, the latter with the use of a new methodology defined as Critical Hydrogen ion concentration Maximum (CHM). In increasing temperatures from 20°C to 37°C, both *Girardia tigrina* and *Girardia* sp. proved to be adaptable to thermal stress. *Girardia* sp. was shown to be more resistant to higher temperatures. However, *Girardia tigrina* was more resistant to extreme pH conditions (4.0 to 10.0). SEM analysis showed morphological differences among planarian species, such as the arrangement of the structures and cell types of the dorsal epidermis. Moreover, planarians demonstrated the ability to change the surrounding pH of their external environment in order to maintain the function of their physiological mechanisms, suggesting that these animals have a complex survival system, possibly related to protonephridia, flame cells and excretory pores.

This article has an associated First Person interview with the first author of the paper.

## Abstract (precis)

**Summary:** In this scanning electron microscopy study, we report the physiological findings regarding the structures of the epidermis of planarians, before and after exposure to temperature and pH stressors.

## INTRODUCTION

Planarians are found worldwide in a variety of habitats, ranging from humid, terrestrial ecosystems to freshwater and marine saltwater environments (Alvarez-Presas et al., 2008; Solà et al., 2015). Freshwater planarians are metazoan flatworms which are bilaterally symmetrical. They belong to the phylum Platyhelminthes and lack a circulatory system, a respiratory system, and skeletal structures (Knakievicz, 2014; Reddien and Alvarado, 2004). They feed predominantly on insects, insect larvae, and other invertebrates. These animals tolerate feeding deprivation and can survive months without any food at all (Reddien and Alvarado, 2004; Baguña, 2012). They have high plasticity, and they may be kept and manipulated easily in the laboratory (Saló, 2006; Alvarado, 2012).

But what makes them unique among metazoans is their great regenerative capacity. Throughout their body, they have pluripotent stem cells, called neoblasts, which are proliferative cells with the capacity to promote tissue regeneration that fills the space between the epidermis and the intestine, called the mesenchyme or parenchyma (Baguña, 2012; Karami et al., 2015; Adler and Alvarado, 2015). Neoblasts, quantified by Reddien and Alvarado (2004), make up approximately 25% to 30% of all cells of the planarian's body. After suffering injuries, the planarian neoblasts migrate to the site of the lesion and promote the formation of the ‘blastema’ (Reddien and Alvarado, 2004; Alvarado, 2012). Exposure of neoblasts to low temperature argon plasma (LTAP), known as cold plasma, caused acceleration of the regenerative processes after the treatment in *Schmidtea mediterranea* (Benazzi, 1975; Ermakov, et al., 2016).

Different scientific techniques are currently being tested to see whether they can shed light on the molecular pathway of limnic planarian tissue regeneration. Adler and Alvarado (2015) observed the Argonaute Piwi-1 and histone H2B proteins in neoblasts as expressed markers. RNA Interference (RNAi) was carried out on FoxA, EGFR1, PABP-2 and p53 gene transcripts to track defects in the reconstruction of structures by neoblasts (Scimone, et al., 2014; Adler, et al., 2014).

The water temperature is an important factor involved in the distribution and abundance of the different planarian species (Claussen and Walters, 1982). Planarians possess thermo-sensory structures throughout their bodies which are activated by ion channels of the melastatin TRP family (TRPM). They exert this function through their interaction with serotonergic neurons; these structures may be responsible for the thermo taxis exhibited by these animals (Inoue, et al., 2014). The freshwater planarians *Girardia tigrina* (Girard, 1850) and *Girardia dorotocephala* (Woodworth, 1897) were studied on how they adapted to variation in water temperature and thermal acclimatization. They demonstrated that the adaptation and resistance responses of *Girardia tigrina* due to temperature changes, 5°C to 25°C or 25°C to 5°C, were faster compared to those of *G. dorotocephala* (Claussen and Walters, 1982). In 1985, Tsukuda and Ogoshi evaluated three groups of *Dugesia japonica* (Ichikawa and Kawakatsu, 1964), that were acclimatized for 6 months at 13°C, 18°C and 20°C using a platform on which the temperature increased gradually from 9°C to 27°C. The researchers demonstrated that these animals have a preference for the temperature for which they were acclimatized.

Cowles and Bogert, when studying how animals move when under thermal stress, developed the ‘Critical Thermal Maximum’ (CTM or CTMax) parameter that although occurs at different temperatures for different species, exhibits a single behavioral pattern of slowness, immobility, irregular movements or contortions. The adoption of the CTM parameter allowed researchers to evaluate the thermal tolerance of different organisms (Cowles and Bogert, 1944; Lutterschmidt and Hutchison, 1997; Zhang and Kieffer, 2014). Limnic planarians exposed to the CTM, show signs of contortions, sluggishness, and an injured epidermis, losing their ability to move properly before dying (Claussen and Walters, 1982; Tsukuda and Ogoshi, 1985).

Another factor of equal importance for the acclimatization of free-living invertebrates of lentic ecosystems is the hydrogen ion concentration established by its potential of hydrogen (pH) (Feldman and Connor, 1992), which is directly related to the temperature and the ions and salts present in the solution and to its carbon dioxide tension (Powers, 1930). Aquatic animals are able to withstand wide pH variations by regulating their organs internally, although it might have its physiology and behavior altered (Powers, 1930; Feldman and Connor, 1992; Rivera and Perich, 1994).

Freshwater planarians secrete metabolic fluids and mucus capable of altering the pH of the environment in which they live. This pH change occurs naturally according to each species of planarians, according to its physiological needs (Vara et al., 2008; Rink, 2013), in relation to the pH values of freshwater sources in its environment, which range between 4.0 and 9.0 around the world (Rivera and Perich, 1994).

Although pH is directly related to the physiology and behavior of planarians, the maximum hydrogen-ion potential of these animals is not known. In this study, we evaluated the adaptive and regenerative capacities of *Girardia tigrina* (Girard, 1850) and *Girardia* sp. undergoing the Critical Thermal Maximum (CTM) and the Critical Hydrogen ion concentration Maximum (CHM), independently.

## RESULTS

### Critical thermal maximum

**Fig. 1.**
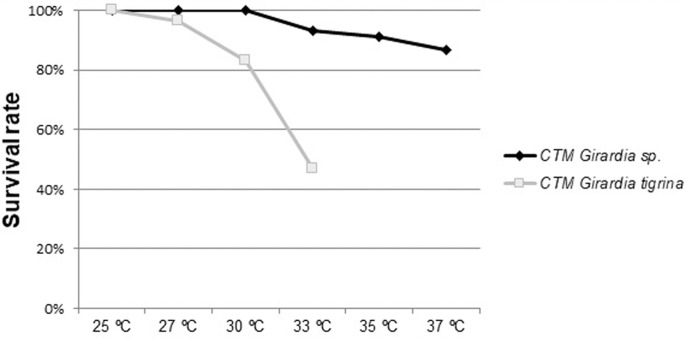
**Average survival rate of CTM groups with 90 specimens of *Girardia* sp. and 90 specimens of *Girardiatigrina* after heat stress.**

All specimens of *Girardia* *tigrina* remained alive at 25°C. With the gradual increase of temperature, specimens of *Girardia* *tigrina* began to die: one death at 27°C (3.34%), four at 30°C (13.33%) and 11 at 33°C (36.67%), totaling 16 deaths (53.34%). In contrast, all specimens of *Girardia* sp. remained viable at 25°C, 27°C and 30°C. There were two deaths at 33°C (6.67%), one at 35°C (3.34%) and one at 37°C (6.67%), totaling four deaths (13.34%) as shown in Fig. 1. All specimens from the control group (maintained at 20°C) remained alive throughout the experiment. These death numbers were based on the average of deaths obtained in the three replicate experiments.

The remaining live specimens of both species, *Girardia* *tigrina* at 33°C and *Girardia* sp. at 37°C, at this time, presented slow movements, release of mucus and metabolic fluids, and an injured epidermis. The behavioral changes that preceded the deaths were similar for both species and started after exposure to a temperature higher than 30°C.

At each temperature observation, the maintenance water pH (6.3±0.1) of all groups was evaluated showing an average of pH 6.4 to 7.5. The control was just maintenance water (Table 1).

**Table 1.**
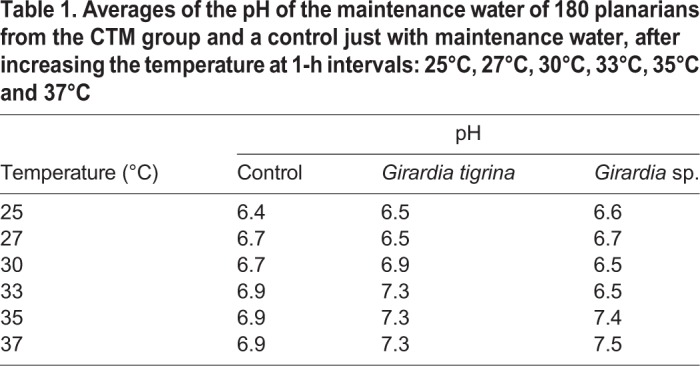
**Averages of the pH of the maintenance water of 180 planarians from the CTM group and a control just with maintenance water, after increasing the temperature at 1-h intervals: 25°C, 27°C, 30°C, 33°C, 35°C and 37°C**

### *Girardia tigrina* and *Girardia* sp. epidermis

**Fig. 2.**
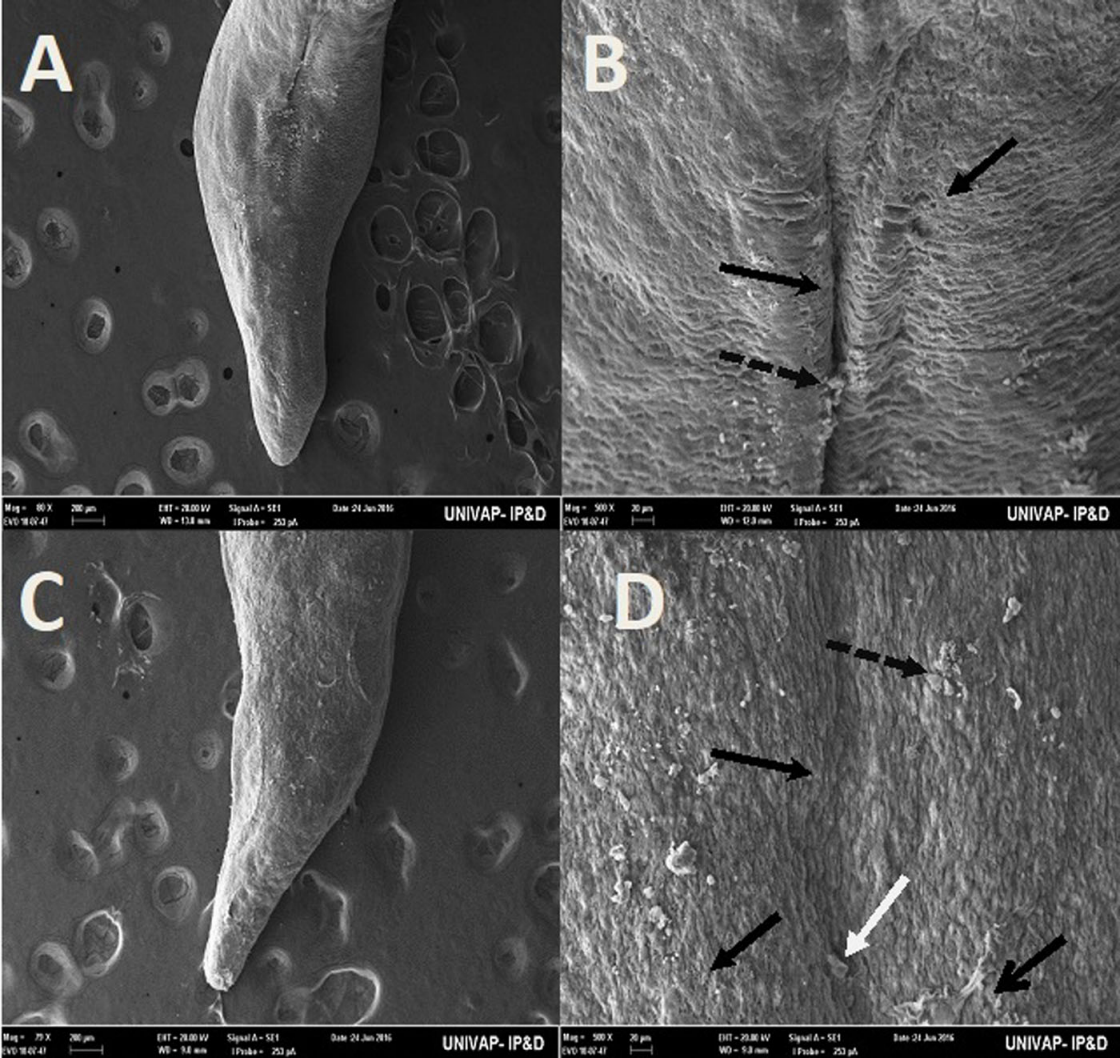
**Scanning electron microscopy of *Girardia* sp. (A,B) and *Girardiatigrina* (C,D) from the control group.** (A,C) Dorsal epidermis (80×). (B) Invagination on epidermis (anterior-central region) (500×) showing presence of secretory droplets and excretory pores. (D) Invagination on epidermis (anterior-central region) (500×) showing presence of secretory droplets, excretory pores, a little rhabdite and mild formation of mucus in the lower part of the planarian body. General characteristics observed in ten specimens.

**Fig. 3.**
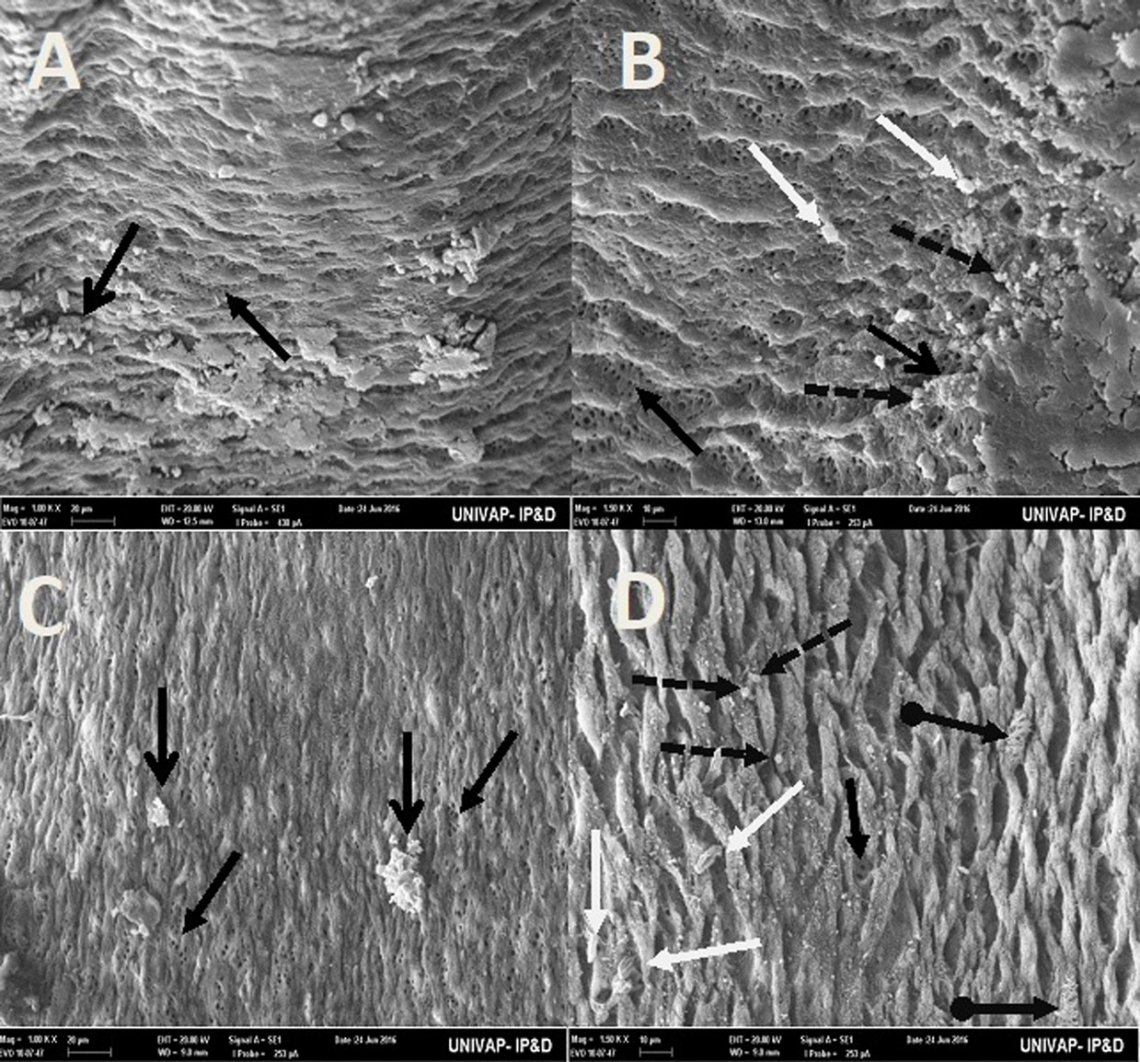
**Scanning electron microscopy of *Girardia* sp. (A,B) and *Girardiatigrina* (C,D) from the control group.** (A,B) (100k× and 150k×, respectively) Horizontal arrangement of the cells of the upper dorsal epidermis and excretory pores of *Girardia* sp., showing presence of secretory droplets, little rhabdites and poor mucus formation. (C,D) (100k× and 150k× respectively) Vertical arrangement of cells of the upper dorsal epidermis and excretory pores of *Girardia* *tigrina*, showing presence of secretory droplets, a greater number of rhabdites, mucus formation and hair cells (ciliary). General characteristics observed in ten specimens.

**Fig. 4.**
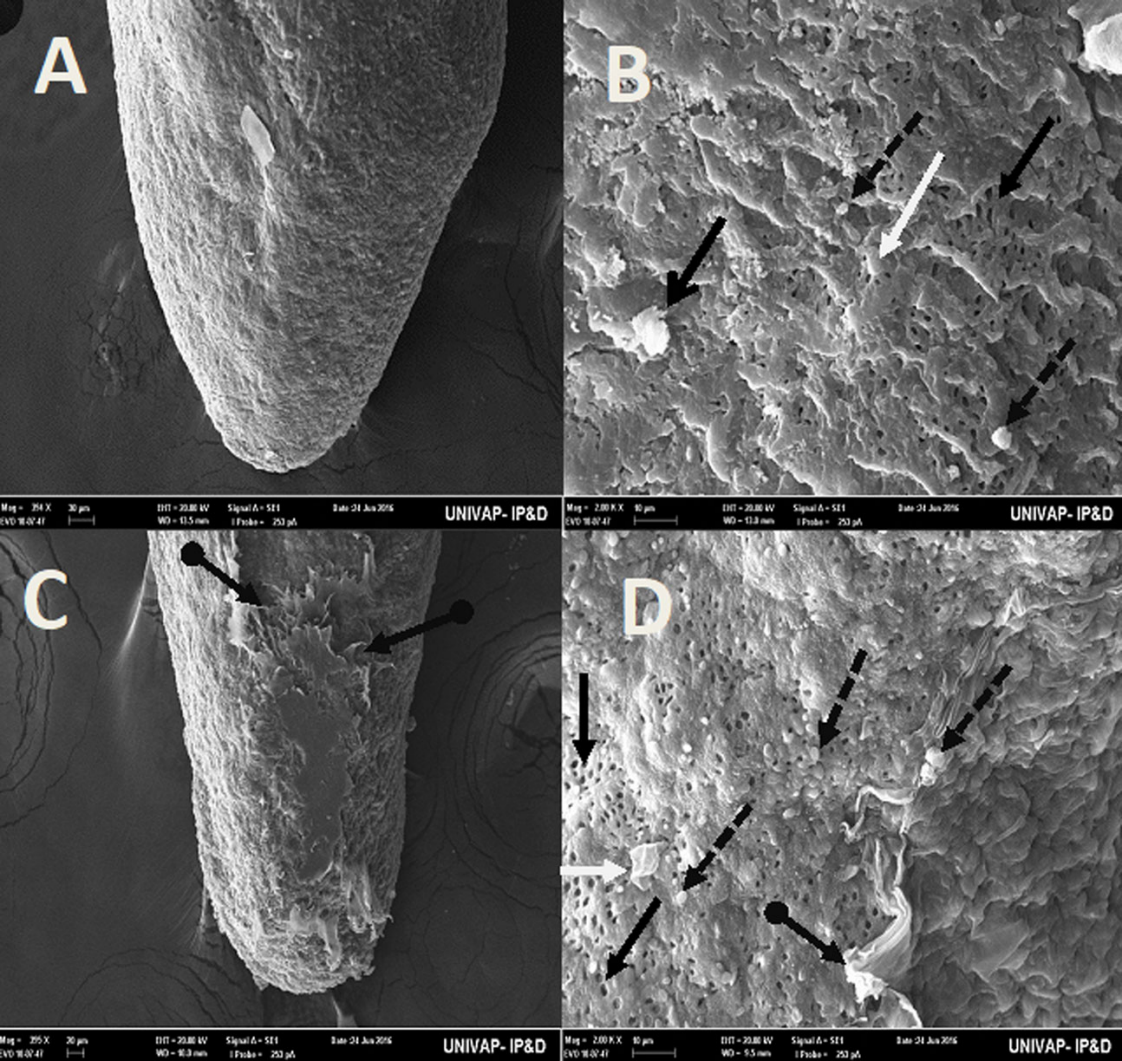
**Scanning electron microscopy of *Girardia* sp. (A,B) and *Girardiatigrina* (C,D) from the control group.** (A,C) (395×) End of the posterior region (tail). (B) (200k×) Presence of secretory droplets, excretory pores, rhabdites and mild mucus formation. (D) (200k×) Presence of secretory droplets, excretory pores, rhabdites, hair cells (ciliary) and mild mucus formation. General characteristics observed in ten specimens.

Table 2 may be used to better understand the images taken of the epidermis of specimens from the CTM, CHM, and control groups. Specimens from the control groups of *Girardia* sp. and *Girardia* *tigrina*, showed dorsal epidermis structures such as excretory pores, mucus formation, little presence of hair cells, rhabdites and secretory droplets (Figs 2–4). On the dorsal side of the planarians, the formation of an invagination, near the anterior region, below the auricles (Fig. 2), and the arrangement of cell tissue of the upper epidermis and excretory pores (Fig. 3), were observed. Fig. 4 highlights the difference observed at the end of the posterior region (tail) of both species in terms of the presence of hair cells (ciliated structures).

**Table 2.**
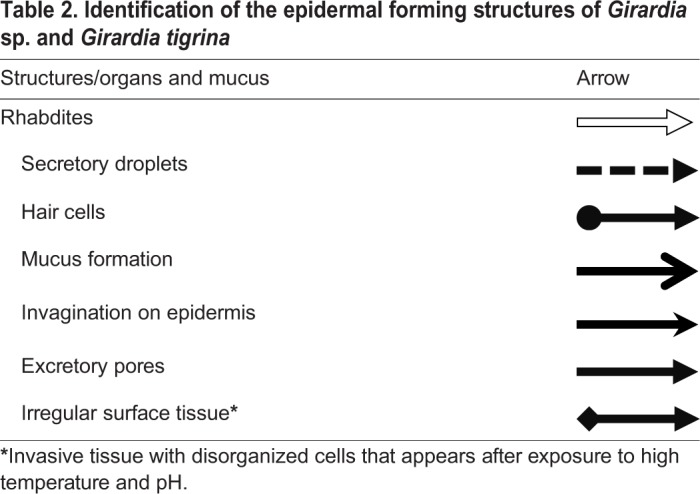
**Identification of the epidermal forming structures of *Girardia* sp. and *Girardiatigrina***

Table 3 summarizes the main morphological differences found between *Girardia tigrina* and *Girardia* sp., in terms of the arrangement of the cells of the upper dorsal epidermis (horizontal or vertical), the presence or absence of cilia structures at the end of the posterior region of the animal, and the presence and size of invagination on the anterior epidermis (below the auricles).

**Table 3.**
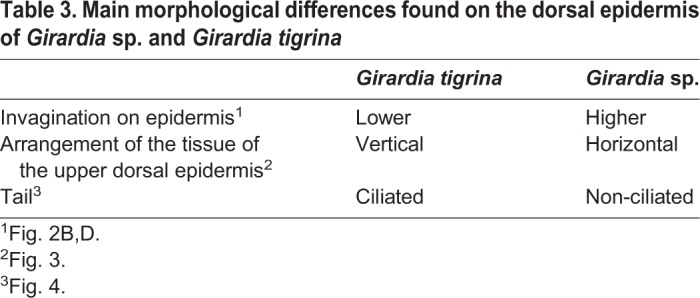
**Main morphological differences found on the dorsal epidermis of *Girardia* sp. and *Girardia tigrina***

### *Girardia tigrina* and *Girardia* sp. epidermis alterations after heat stress

**Fig. 5.**
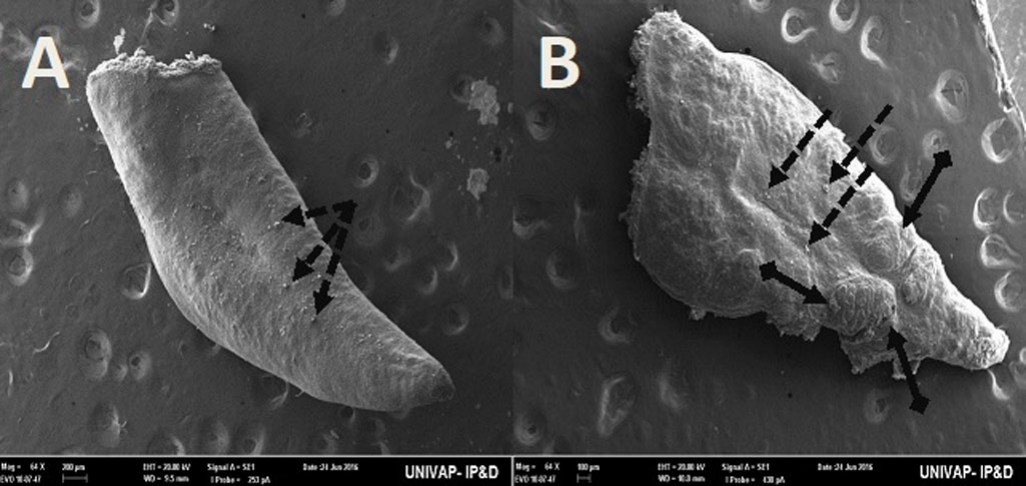
**SEM of epidermis of the specimens from the CTM groups of both *Girardia* sp. (A) and *Girardiatigrina* (B).** (A) (68×) Dorsal view of *Girardia* sp., showing secretory droplets of mucus and granules throughout the planarian's body. (B) (68×) Dorsal view of *Girardia* *tigrina* showing many deformations and irregular structures throughout the body, especially in the post-pharyngeal region, with presence of secretory droplets. General characteristics observed in five specimens.

**Fig. 6.**
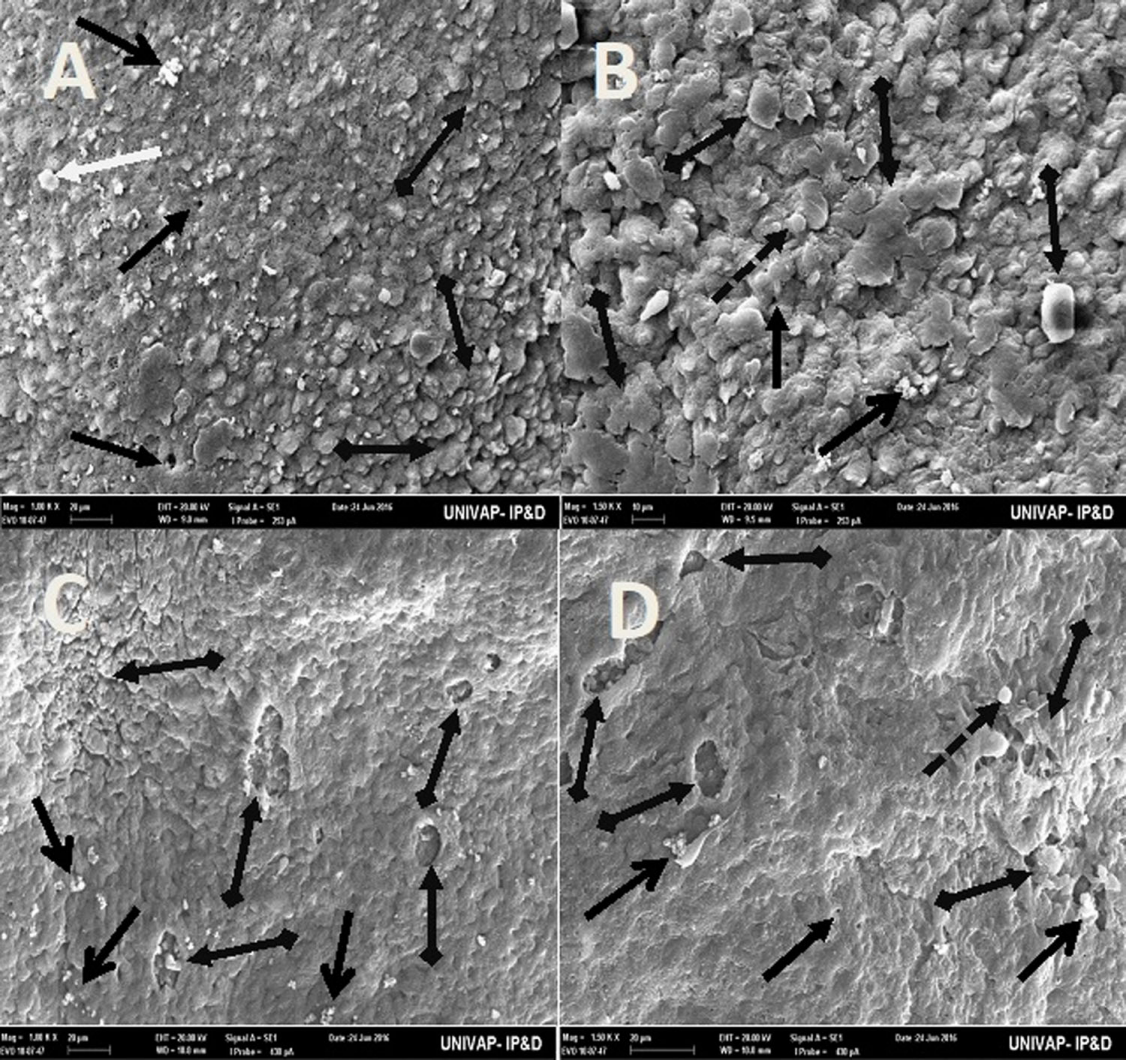
**SEM of epidermis of the specimens from the CTM groups of both *Girardia* sp. (A,B) and *Girardiatigrina* (C,D).** (A,B) (100k× and 150k× respectively) Arrangement of the cells of the upper dorsal epidermis of *Girardia* sp., showing absence of active excretory pores and replacement by an irregular surface tissue presenting many disorganized cells, disorganization of secretory droplets and slight formation of mucus. (C,D) (100k× and 150k× respectively) Arrangement of the cells of the upper dorsal epidermis of *Girardia* *tigrina*, showing few excretory pores, all inactive, and very few secretory droplets, presence of slits with irregular cells grouped in the interior, absence of cilia structures, and formation of an overlapping tissue (invasive tissue) that takes the place of all normal structures. General characteristics observed in five specimens.

**Fig. 7.**
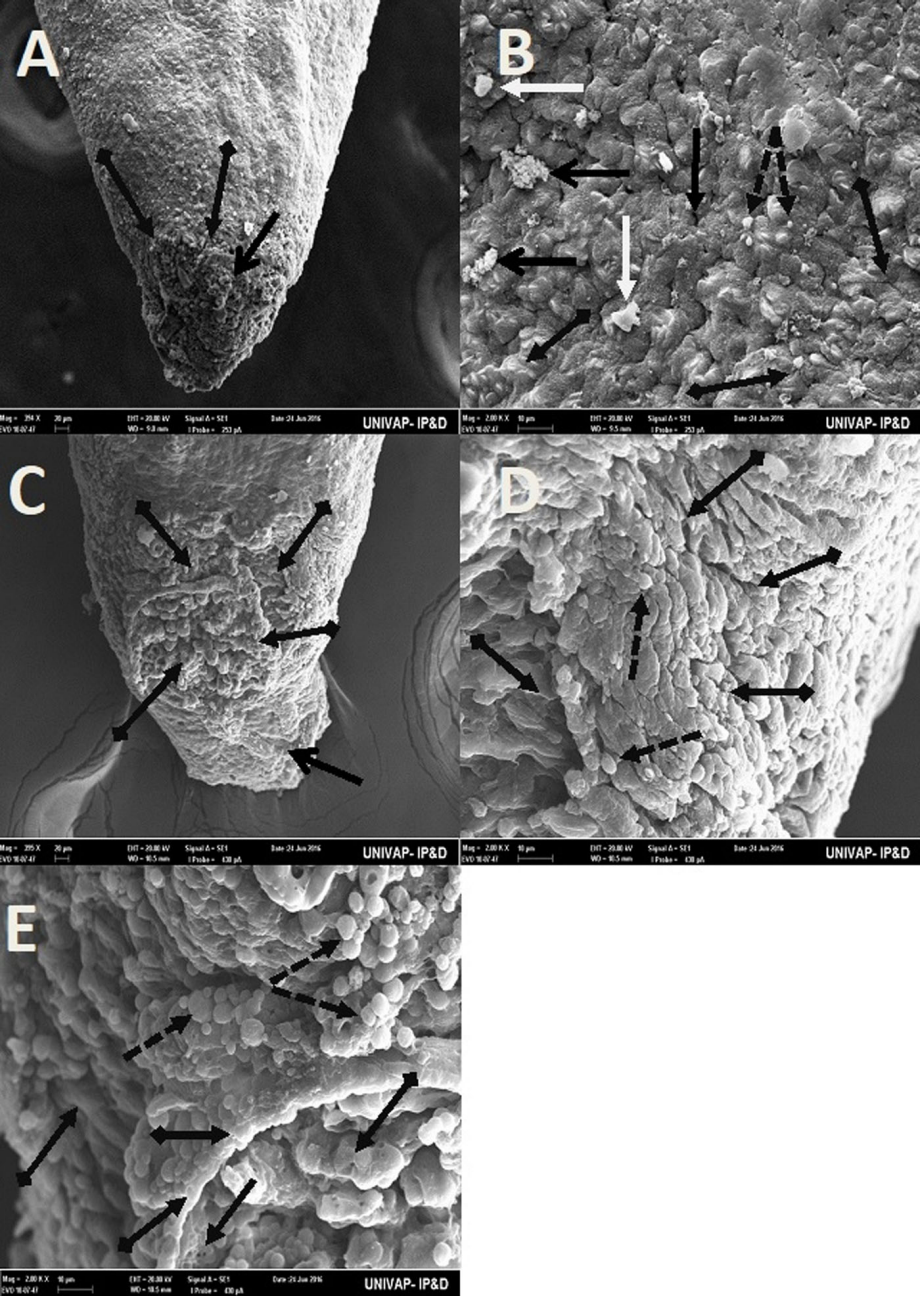
**SEM of epidermis of the specimens from the CTM groups of both *Girardia* sp. (A,B) and *Girardiatigrina* (C-E).** (A,B) (395× and 200k× respectively) View of the end of the posterior region (tail) of *Girardia* sp. showing absence of active excretory pores and replacement by an irregular surface tissue, presenting many disorganized structures such as secretory droplets, a shrunken rhabdite and the presence of lumps of mucus. (C-E) (395k×, 200k× and 200k× respectively) View of different areas of the end of the posterior region (tail) of *Girardia* *tigrina* showing a non-characteristic and disorganized tissue with many deformations (C,D), atypical enlarged secretory droplets of mucus grouped in clusters (E), very few inactive excretory pores and an absence of rhabdites and hair cells (cilia). There were formations of mucus lumps and atypical cells and structures (C-E). General characteristics observed in five specimens.

Specimens from the CTM group of *Girardia* *tigrina* and *Girardia* sp., assessed by SEM, revealed alterations in structures of the dorsal epidermis; specifically regarding the organization and integrity of excretory pores, hair cells, rhabdites, and secretory droplets (Figs 5, 6 and 7). The two species exhibited differences in resistance to critical temperature. The epidermis of the *Girardia tigrina* specimen was more sensitive, showing more damage to the structures of the epidermis than those seen in the *Girardia* sp. specimen.

Both *Girardia* *tigrina* (33°C) and *Girardia* sp. (37°C) specimens suffered epidermal damage, especially the tissue of the end of the posterior region (tail) (Fig. 7).

### Effects of the critical hydrogen ion concentration maximum (CHM)

Specimens of *Girardia* *tigrina* and *Girardia* sp. in the CHM group were kept at a constant temperature of 20°C, while the pH of their maintenance water was adjusted to 3.0, 4.0, 5.0, 6.0, 7.0, 8.0, 9.0, and 10.0. Both species of planarians incubated at pH 3.0 presented contortions and mucus release, dying between 5 and 15 min after exposure and, therefore, were not used in the data analysis. After 1 h of exposure, both species incubated in 4.0 and 10.0 pH developed a swollen body structure, released mucus, and moved slowly or remained immobile.

All specimens from both species maintained at 5.0, 6.0, 7.0, 8.0 and 9.0 pH showed no visible changes or atypical behavior after 3 h and 18 h of incubation. Specimens of *Girardia tigrina* laid cocoons at pH 7.0.

The measurements of the pH were carried out at the beginning of the experiment and after 3 h and 18 h specifically to verify the pH adaptability of these animals (*F*=6.0000, *P*<0.01) by species as shown in Table 4.

**Table 4.**
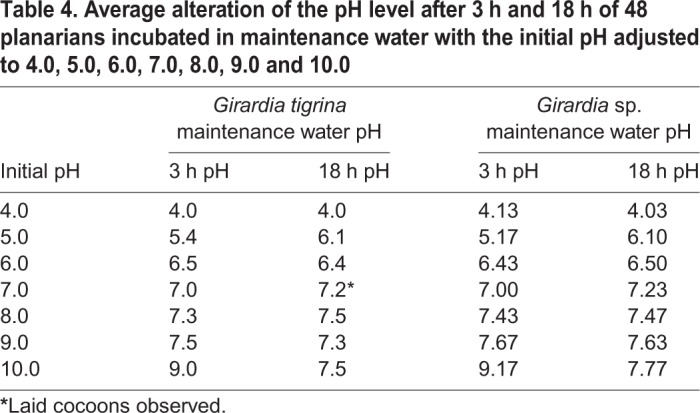
**Average alteration of the pH level after 3 h and 18 h of 48 planarians incubated in maintenance water with the initial pH adjusted to 4.0, 5.0, 6.0, 7.0, 8.0, 9.0 and 10.0**

The variations in the measurements of the pH level of the incubation water of both species (*F*=399.1184, *P*<0.01) were also analyzed, at the beginning (0 h) and 3 h and 18 h later. Both species were able to acidify or to alkalize the pH of the external medium, suggesting adaptation to physiological needs (Table 5).

**Table 5.**
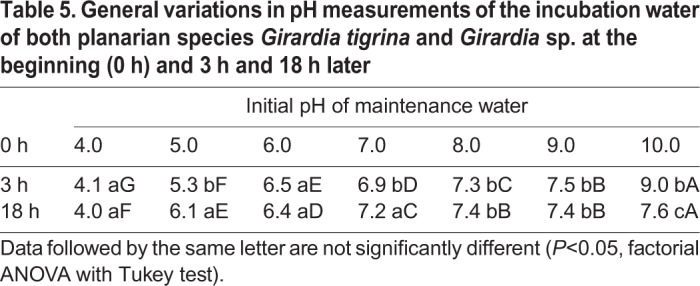
**General variations in pH measurements of the incubation water of both planarian species *Girardiatigrina* and *Girardia* sp. at the beginning (0 h) and 3 h and 18 h later**

### *Girardia tigrina* and *Girardia* sp. epidermis after CHM

**Fig. 8.**
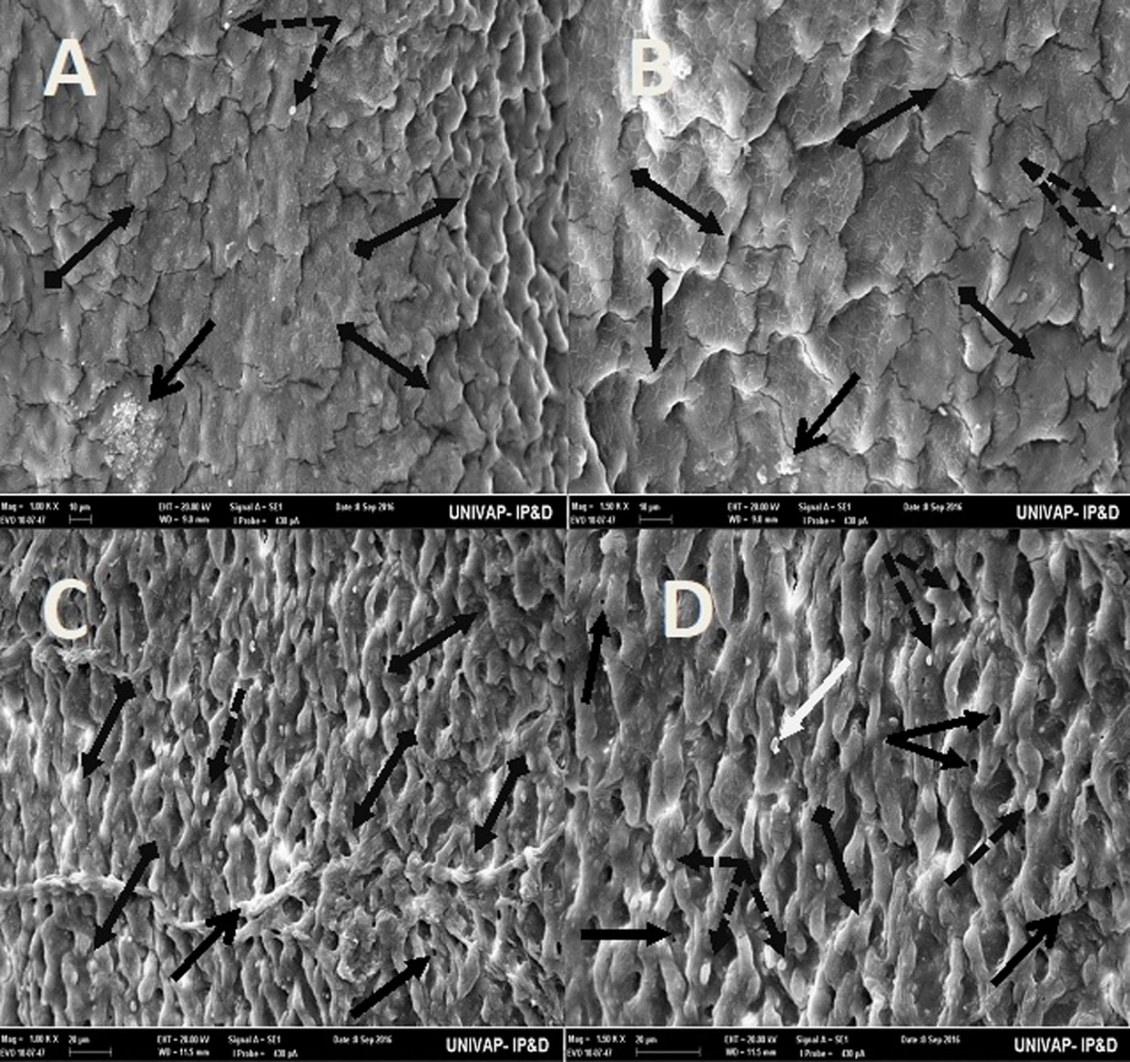
**Scanning electron microscopy of *Girardia* sp. (A,B) and *Girardiatigrina* (C,D) exposed for 18 h in maintenance water with the pH adjusted to 4.0.** (A,B) (100k× and 150k× respectively) Arrangement of the structures in upper dorsal epidermis of *Girardia* sp., showing absence of excretory pores and cilia and presenting an irregular surface tissue with atypical cells that took the place of all other structures. Intense abnormal activity of secretory droplets of reduced size and mucus formation at the bottom of the images. (C,D) (100k× and 150k× respectively) Upper dorsal epidermis of *Girardia* *tigrina* showing few active excretory pores, disorganization of secretory droplets of reduced size with intense activity releasing mucus, presence of slits with irregular cells grouped in the interior, absence of hair cells, a single rhabdite of small proportions and probably inactive. Formation of an overlapping regular surface tissue, better structured than in *Girardia* sp. (A,B). General characteristics observed in five specimens.

**Fig. 9.**
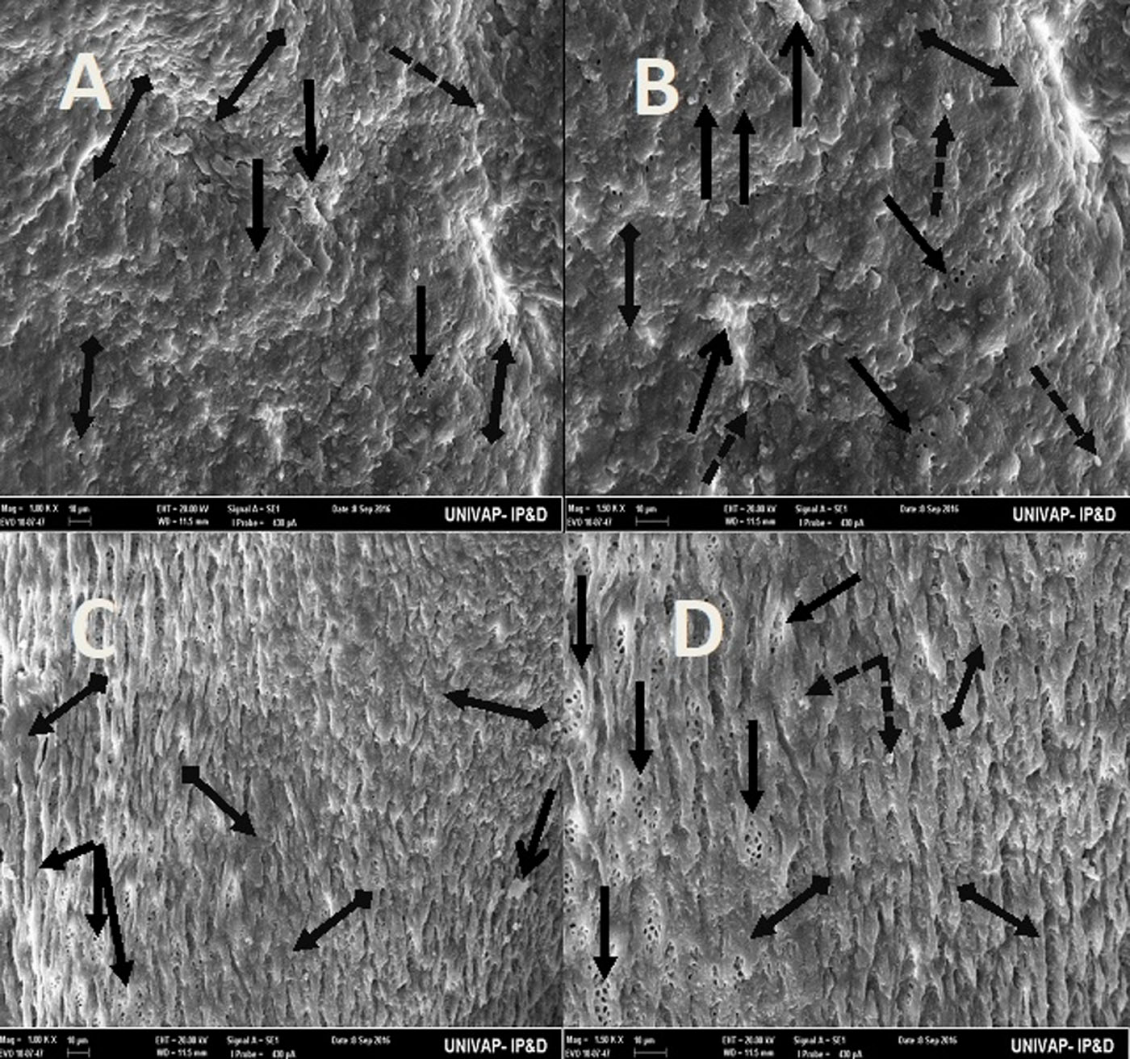
**Scanning electron microscopy of *Girardia* sp. (A,B) and *Girardiatigrina* (C,D) exposed for 18 h in maintenance water with the pH adjusted to 10.0.** (A,B) (100k× and 150k× respectively) Arrangement of the structures in upper dorsal epidermis of *Girardia* sp., with moderate presence of active excretory pores and replacement by irregular surface tissue in a few areas, intense activity of secretory droplets with mucus formation. (C,D) (100k× and 150k× respectively) Upper dorsal epidermis of *Girardia* *tigrina*, with active excretory pores of common appearance, few secretory droplets and little mucus formation. Absence of hair cells and presence of overlapping regular surface tissue in the upper right corner (C). General characteristics observed in five specimens.

The *Girardia* sp. and *Girardia* *tigrina* from the CHM group were incubated for 18 h in maintenance water with the pH adjusted to 4.0 or 10.0 and were analyzed in SEM (Figs 8 and 9). The dorsal epidermis-forming structures, such as the arrangement, organization and integrity of excretory pores, hair cells, rhabdites and secretory droplets were evaluated to determine the effects of the pH on the epidermis of both species.

## DISCUSSION

The present study evaluated the physiological effects of the exposure of the freshwater planarians, *Girardia tigrina* and *Girardia* sp. to heat and pH stress. In addition, this paper provides information about the physiological structures of the epidermis of a specimen under study as a new species, *Girardia* sp., in comparison with the epidermis of *Girardia tigrina*.

Specimens of both species were adaptable to thermal stress at increasing temperatures. *Girardia* sp. is a species native to Brazil and showed to be more resistant to higher temperatures than *Girardia* *tigrina*, which may be a local climatic adaptation characteristic of this tropical Brazilian species, since *Girardia* *tigrina* is a globalized species (Vara et al., 2008). The thermal adaptive capacity of *Girardia* *tigrina* was described by Claussen and Walters (1982) and later by Rivera and Perich (1994).

In extreme temperatures, the physiological events that preceded the deaths of individuals of the two species of planarians were similar to those reported by other researchers; just as with the *Ictalurus punctatus* fish, they presented spasms and loss of balance in response to CTM when exposed to the temperature oscillation of 23°C to 33°C in 24-h cycles (Díaz and Bückle, 1999).

The CTM parameters established by Cowles and Bogert (1944) and more widely discussed by Lutterschmidt and Hutchison (1997) are considered effective to evaluate the physiological behavior of different aquatic and non-aquatic animals during thermal stress. Other ectothermic organisms presented similar responses to heat stress, as described by Vinagre et al. (2015), such as the *Bathygobius soporator* and *Parablennius marmoreus* fish species; the *Palaemon northropi* and *Hippolyte obliquimanus* shrimp; the *Eurypanopeus abbreviatus* and *Menippe nodifrons* crabs. These animals were removed from their natural habitat, acclimatized in a controlled environment for 7 days and deprived of food for 24 h prior to the experiments. In the shrimp and fish, the authors observed loss of balance, corroborating the findings with the *Ictalurus punctatus* fish described by Díaz and Bückle (1999) and with the results presented in this study for the species of planarians *Girardia tigrina* and *Girardia* sp. All these data together point to the efficacy of the CTM method as a physiological evaluation parameter for different organisms subjected to thermal extremes.

The analysis of the epidermis of the two species of planarians by SEM showed significant morphological differences (Table 3) as can be seen in the arrangement of the structures and cell types of the dorsal epidermis, vertically disposed in *Girardia* *tigrina*, but horizontally exposed in *Girardia* sp. (Fig. 3). This finding differs from that observed with *Dugesia tigrina* from Smales and Blankespoor (1978), who described it as irregularly contoured. Another different point was the presence of a remarkable invagination on the epidermis of the anterior region and below the auricles in *Girardia* sp. (Fig. 3B), while *Girardia* *tigrina* presented only a slight depression (Fig. 2D). The end of the posterior region (tail) of the planarians was also different, presenting cilia in *Girardia* *tigrina*, but not in the tail of *Girardia* sp. (Fig. 4A,C). Ciliated structures were not abundantly visualized in this study since they are structures commonly found in the ventral surface of *Girardia* *tigrina* and on the dorsal surface, especially in areas such as the auricles, although they can also be observed in the dorsal epidermis of other species (Smales and Blankespoor, 1978).

The end of the posterior region of these animals proved to be the most vulnerable area to heat and pH alterations. *Girardia* *tigrina* was more susceptible to heat stressors than *Girardia* sp., exhibiting a tissue with many deformations composed of mucus lumps distributed through slits filled with atypical cells in the epidermis of the affected region (Fig. 7) and in more than 50% of deaths until a 33°C incubation temperature (Fig. 1).

New overlapping tissue took the place of the normal physiological structures in the dorsal epidermis of both planarian species exposed to extreme temperatures or pH values: *Girardia* *tigrina* (33°C) and *Girardia* sp. (37°C) (Figs 6 and 7) and at pH 4.0 and 10.0 (Figs 8 and 9). The tissue was composed of irregular structures; there were few secretory droplets and fewer rhabdites. The absence of rhabdites and mucus on the epidermis of both planarians can be explained by the deterioration or inactivation of the excretory pores, which are responsible for the excretion of the mucus produced by the rhabdites. These excretory cells act by forming and accumulating mucus which releases it upon rupture through the excretory pores on the surface of the epidermis. The mucus has a protective function and is released in response to stress (Bowen and Ryder, 1974; Smales and Blankespoor, 1978). The absence of this response mechanism explains the difficulty in locomotion of animals of both species observed during CTM and CHM as described in the results.

After being exposed to extreme heat stress (33°C: *Girardia* *tigrina*, 37°C: *Girardia* sp.) and extreme pH (4.0 and 10.0) both species also presented affected (disorganized) secretory droplets (Figs 7 and 8). These results corroborate those described by Smales and Blankespoor (1978) that reported that the distribution of the secretory droplets of substances, above the excretory pores, are common and have a protective activity, similar to the rhabdites.

In this study it was possible to observe that the two species of planarians, *Girardia tigrina* and *Girardia* sp., were able to change the potential of hydrogen (pH) of the external environment in order to maintain the functioning of their physiological mechanisms (*F*=6.0000, *P*<0.01) (Table 4), corroborating the results of Rivera and Perich (1994), who stated that limnic planarians were able to secret metabolic fluids and mucus, promoting the adaptation of the pH of the external environment to their needs.

The specimens assessed with the CHM survived 18 h of exposure in maintenance water with pH adjusted from 4.0 to 10.0. The exposures at pH 3.0 caused the death of the specimens within 15 min. These findings indicate the inability of species to adapt to this pH level, probably due to the inefficiency of the physiological mechanisms of mucus and fluid release.

The survival of *Girardia* *tigrina* in pH between 4.0 and 9.0 and death of this species at pH 3.0, had already been described by Rivera and Perich, 1994, using asexual specimens. The present study, besides reaffirming the observations made by the authors, showed the capacity of sexual reproduction of *Girardia* *tigrina*; they laid cocoons between 3 h and 18 h of exposure at pH 7.2±0.1 (Table 4), as well as the adaptation ability (*F*=399.1184, *P*<0.01) observed in both species in 3 h and 18 h at initial pH 10.0 (Table 5).

The *Girardia* sp. demonstrated the same adaptive skills as *Girardia* *tigrina* and could not survive at pH 3.0, corresponding with the observations made by Rivera and Perich, 1994, in *Girardia dorotocephala* (Woodworth, 1897), *Cura foremanii* (Girard, 1852), *Dendrocelopsis vaginatus* (Hyman) and asexual specimens of *Girardia tigrina* (Girard, 1850).

The observation of the epidermis through scanning electron microscope of both planarians carried out after 18 h from the beginning of exposure to pH 4.0 and 10.0, revealed physiological damage such as the absence of excretory pores and a lack of fundamental structures due to the presence of an overlapping tissue (Figs 8 and 9). However, both species were able to change the pH of the maintenance water, being more efficient in acidifying than basifying (alkalize) the environment. In addition, the planarians demonstrated a preference in balancing the pH of the medium between 7 and 7.5 (Table 5), which differed from the maintenance water's pH of 6.3±0.1.

The planarians exposed to pH 4.0 had the epidermis more damaged in comparison to those exposed to a basic pH. At pH 10.0, the epidermis of the specimens presented a greater number of functional structures, such as more active excretory pores and secretory droplets, less presence of overlapping tissue and few protective mucus on the epidermis (Figs 8 and 9).

After submitting both species to the CTM or CHM, no more ciliated structures/hair cells were found in the dorsal epidermis of the animals (Figs 5–9), indicating severe damage, since these delicate structures act as photoreceptors and sensory neurons as described in *Schmidtea mediterranea* by Lapan and Reddien (2012). Cilia and/or hair cells can also have a mobility function as described for *S.* *mediterranea*, but they are most commonly found in areas of the ventral surface (surface of the pharynx). They can also be seen composing the protonephridia, a type of fluid excretory system formed by tubules that act by osmoregulation ending at the surface of the planarian. This system is composed by a set of cells called flame cells (Basquin, et al., 2015). The planarian protonephridia is currently considered a model for the study of renal diseases due to the similarities it presents with mammalian nephrons (Issigonis and Newmark, 2015).

The results presented in this study suggest that the symptoms observed in the planarians after exposure to CTM and CHM, characterized as end-point criteria (contortion or slow movements, abnormal release of mucus and metabolic fluids, injured epidermis and swollen body) or events that predate death according to Cowles and Bogert (1944), are related to the disruption of the osmotic balance of the animal's body. The heat and pH alterations induced the formation of an overlapping tissue that covered the excretory pores of the epidermis and destroyed the rhabdites, probably by promoting the swelling of the animals before their death. The excretory pores together with flame cells distributed along the dorsal epidermis of the planarians, including the head region, acts on the osmotic regulation of the animal and consequently on the functioning of its homeostasis (Nakamura, et al., 2014).

Different from that observed for the CTM, the findings observed in the CHM group revealed that *Girardia tigrina* was more resistant to extreme pH than *Girardia* sp. Images of the epidermis of the two species showed a great amount of active excretory pores (Figs 8 and 9), better structured organs and less presence of overlapping tissue with disorganized structures in *Girardia* *tigrina*. As a cosmopolitan species, commonly found in freshwater sources ranging from pH 4.0 to 9.0 in Europe, North America, and South America, and more rarely in other localities (Vara et al., 2008), *Girardia tigrina* may have developed wide adaptability at different pH values (Rivera and Perich, 1994).

In a confined space, like a vessel under laboratory conditions, planarians can totally change the pH of the water. In their natural environment planarians will not be able to change the pH around them but may be able to withstand variations until reaching their ‘limit-point’; that is, the maximum pH level in which their excretory system can still function. The planarian's microenvironment is basically aquatic plant roots. Given that Planarians start to adapt their physiological organs to influence their microenvironment and, in this way, adapt themselves to live inside that space. If the animal cannot dispose of an internal adaptation suitable to its environmental conditions, it will begin to suffer alterations in its morphology, and reproduction, and die until the planarian species disappears completely.

The effects of the CTM upon freshwater planarians, *Girardia tigrina* and *Girardia* sp. revealed that both are adaptive. The protocol described for CTM was effective for the behavioral evaluation of these animals. The CHM was also effective for assessing the adaptive and reproductive capacities of animals exposed to extreme pH values. They were able to alter the pH of the external environment, probably by means of the secretion of metabolic fluids, in order to withstand and survive. Upon analyzing the CTM and CHM effects on the planarians, it seems that these animals probably exhibit a survival system related to protonephridia, flame cells and excretory pores.

This study opens up new questions and directions for future technological and scientific approaches for the study of the physiology of freshwater planarians in relation to pH and temperature.

## MATERIALS AND METHODS

### Location of the study

The study was carried out in the Laboratory of Planarians (LaPla) in association with the Laboratory of Cellular and Molecular Biology of Fungi, at the Research and Development Institute of the University of Vale do Paraíba, São José dos Campos, São Paulo, Brazil.

### Selection and cultivation of planarians

We selected 207 specimens of *Girardia* sp. and of *Girardia tigrina*. They were in perfect morphological condition with a size ranging from 0.8 cm to 1 cm. They were placed individually into plastic vessels (5 cm diameter×7 cm height) containing 10 mL of maintenance water which was free of chlorine and chemical agents. The pH at the source was 6.3±0.1, collected in the city of Jacareí, São Paulo, Brazil (23°12′38.2″S 45°57′56.6″W).

Before the experiments were run the specimens were deprived of food for 15 days, according to the protocol used for experiments with planarians (Garcia-Fernàndez, et al., 1991; Cebrià et al., 2002; Souza, et al., 2005). The animals were then distributed between the CTM (60 individuals) and the CHM (9 individuals) groups.

### Critical thermal maximum (CTM)

Sixty specimens of *Girardia* *tigrina* and *Girardia* sp. were divided into control and experimental groups (30 of each specimen per group), then placed inside acclimatized incubators (model MA 415, Marconi, São Paulo, Brazil) in the dark, at 20°C for 48 h for pre-acclimatization, according to the protocol of Rivera and Perich (1994). The specimens were observed at 3 h intervals. Next, the temperature was raised 1°C every hour and the animals were observed at temperatures of 25°C, 27°C, 30°C, 33°C, 35°C and 37°C. The parameters used to evaluate the CTM effects were: death, contortions, slowness and damaged epidermis. A Zoom 2000 stereomicroscope (Leica, Wetzlar, Germany) was used for the observations. The control group remained at the acclimatized constant temperature of 20°C. The pH of the maintenance water of each specimen was measured every hour at all temperatures. This experiment was carried out three independent times.

### Critical hydrogen ion concentration maximum (CHM)

For each of the three experimental CHM groups nine specimens of both planarians *Girardia* *tigrina* and *Girardia* sp. were selected, which were placed individually into plastic 5 cm diameter×7 cm height containers with 30 mL of maintenance water, in which the pH was adjusted with 2 M of NaOH or 1 M of HCl to obtain 3.0, 4.0, 5.0, 6.0, 7.0, 8.0, 9.0 and 10.0 pH values. As a control group, two specimens of each species were kept in maintenance water (pH 6.3±0.1), in the dark at 20°C for 18 h.

Behavioral observations and deaths were assessed every 30 min in the first 3 h and after 18 h of incubation in a Leica Zoom 2000 stereomicroscope. The measurements of the pH adjusted maintenance water were performed in different digital-microprocessor pH meters (model PG1800, Gehaka, São Paulo, Brazil; model MA522, Marconi), after 3 h and 18 h from the beginning of the experiment. For analysis and interpretation of the results, factorial ANOVA with Tukey test (*P*<0.05) was performed with the 7.7 version of ASSISTAT software (Silva and Azevedo, 2016). This experiment was carried out three separate times.

### Scanning electron microscopy (SEM)

SEM analysis of the epidermis of the planarians was carried out on ten specimens from the control group and ten specimens from the experimental groups, after sectioning the post auricular region. The specimens were divided into three groups: the control group, the CTM group (*Girardia* *tigrina*, after 4 h at 33°C and *Girardia* sp., after 4 h at 37°C) and the CHM group (*Girardia* *tigrina* and *Girardia* sp. after 18 h in maintenance water with pH adjusted to 4.0 and 10.0).

Post auricular fragments were fixed in 2% paraformaldehyde and 2.5% glutaraldehyde with a 0.1 M sodium cacodylate buffer (pH 7.2) for 30 min and dehydrated serially in 50%, 70%, 90%, and 100% acetone for 10 min each. Then, the samples were adhered to stubs containing carbon tape and metalized with gold powder. The images were obtained in a scanning electron microscope [model mA10 (Evo), Zeiss, Oberkochen, Germany].

In the CTM experiment, the specimens of *Girardia tigrina* were not exposed to 37°C, due to the LD50 obtained for this species at 33°C.

This study was authorized by the Biodiversity Authorization and Information System (SISBIO) under license number 56314.

## Supplementary Material

First Person interview
