# Supplementary material for: Physiological evaluation of the behavior and epidermis of freshwater planarians (Girardia tigrina and Girardia sp.) exposed to stressors
Source: Biol Open. 2018 Jun 15;7(6):bio029595. doi: 10.1242/bio.029595 (PMC6031348; doi:10.1242/bio.029595)
Supplement: First Person interview [file biolopen-7-029595-s1.pdf]

FIRST PERSON

First person – Matheus Salgado de Oliveira

First Person is a series of interviews with the first authors of a selection of papers published in Biology Open, helping early-career researchers promote themselves alongside their papers. Matheus Salgado de Oliveira is first author on 'Physiological evaluation of the behavior and epidermis of freshwater planarians (*Girardia tigrina* and *Girardia* sp.) exposed to stressors', published in BiO. Matheus conducted the research in this article while a MSc student in the lab of Nádia Maria Rodrigues de Campos Velho at the Nature Research Center, University of Vale do Paraíba, São Paulo, Brazil but is now an invited researcher in the same lab, investigating tissue regeneration, ecotoxicology, animal physiology and molecular biology in freshwater planarians.

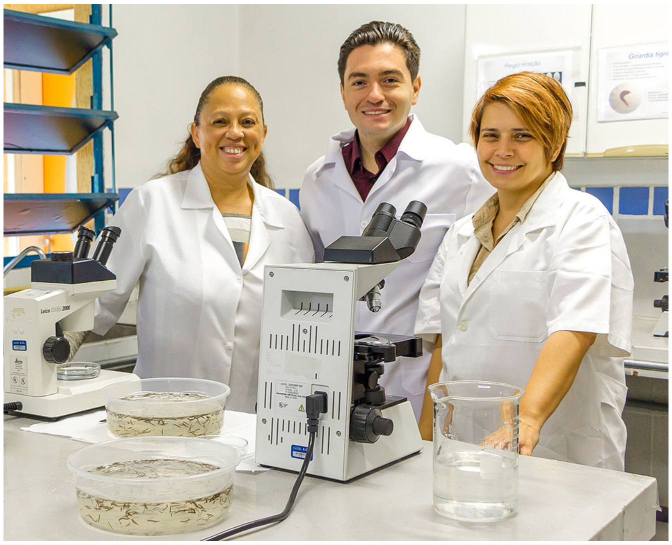

Prof. Nádia Campos Velho, Matheus Oliveira and Prof. Karla Lopes.

What is your scientific background and the general focus of your lab?

I started my research experience at university as a student of a scientific initiation program, and my first steps were in the scientific field of hematology. I graduated with a Bachelor of Biomedicine degree in the biological sciences medical modality. A couple of years later I was introduced to the intriguing scientific area of zoology by Prof. Nádia Maria Rodrigues de Campos Velho. Since I started my MSc in the biological sciences I have developed a passion for this field, especially with freshwater planarians.

These fantastic animals are made up of ~25-30% stem cells and are capable of tissue regeneration after suffering injuries. We have studied a new Brazilian species for the past four years, which is in the characterization phase currently referred to as *Girardia* sp., and have been comparing it with another species that we possess in the Planarian Laboratory (LAPLA), the well-known *Girardia tigrina*. We are studying these animals under different scientific parameters such as animal physiology, taxonomy, molecular biology and ecotoxicology, always aiming for elucidation of the phenomenon of tissue regeneration as our main objective.

> **"Since I started my MSc in the biological sciences I have developed a passion for this field, especially with freshwater planarians."**

How would you explain the main findings of your paper to non-scientific family and friends?

Understanding how freshwater planarians behave in response to different environmental stimuli is to advance the understanding of tissue regeneration and even the quality of the environment. This animal can be used as a bioindicator of freshwater quality. The water temperature and the pH of ecosystems in still fresh water are important factors in the distribution and abundance of several aquatic animals. Although pH is directly related to the physiology of planarians, their adaptive and regenerating capacities under different pH environments was previously unknown. My aim was to observe precisely this question from a scientific point of view and to compare the responses of planarians to variations in pH with their responses to extreme temperatures. I have attempted to describe in detail the physiological structures of two planarian species, comparing *Girardia* sp. to *Girardia tigrina* throughout all procedures. The laboratory of which I am a member is multidisciplinary and cooperative, and our greatest aim is to undertake science with freshwater planarians as the main research model.

What are the potential implications of these results for your field of research?

The primary objective of this study was to observe physiological structures of the epidermis of two different species of planarians through scanning electron microscopy (SEM) and to carefully detail whether these structures and the properties they possessed were similar or not between the two species. The secondary objective was to use critical thermal maximum (CTM) to evaluate the thermal tolerance and the physiological parameters of these animals, and to use SEM to observe alterations in the structures of the epidermis after thermal stress. At this point we asked ourselves if increases in temperature altered the pH of the water, and what effect the pH had on freshwater planarians. Research into freshwater planarians exposed to different levels of pH is scarce in scientific literature, and so their potential for adaptation and their tolerances for these environments were unknown. As it was necessary to create a new methodology, I relied on CTM as a model to evaluate physiological parameters such as tissue regeneration, tolerance, reproductive capacity and epidermal structures, and to investigate the behavior of planarians exposed to different pH values.

What has surprised you the most while conducting your research?

Several findings surprised me in the course of this study. For instance, both planarian species presented morphological

Matheus Salgado de Oliveira's contact details: LAPLA, University of Vale do Paraíba (UNIVAP), São José dos Campos, São Paulo 12244-000, Brazil.

E-mail: matheus.biosciences@gmail.com

Biology Open (2018): doi:10.1242/bio.029595: Supplementary information

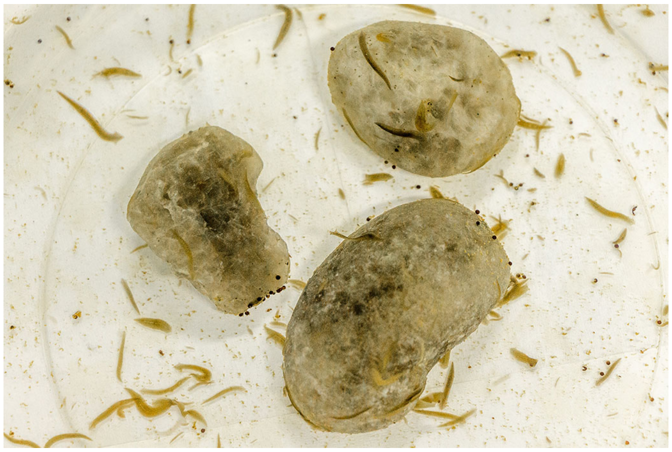

Girardia tigrina planarians. The black dots on the stones are planarian eggs, referred to as cocoons.

differences concerning the arrangement of the structures and cell types of the dorsal epidermis. *Girardia* sp. is a specimen under study as a new species and nothing was previously known about this animal. Both planarian species were adaptable to thermal stress at increasing temperatures. Comparisons between SEM images of the animals before and after stress tests with different pH values were also very interesting and rich in information. However, the most striking finding for me was the capacity of both planarian species to change the pH of their external environment, demonstrating a preference for balancing the pH of the medium between 7.0 and 7.5, even with a starting point as extreme as a pH of 10.0, for example.

> "[…] the most striking finding for me was the capacity of both planarian species to change the pH of their external environment"

What, in your opinion, are some of the greatest achievements in your field and how has this influenced your research?

To control the phenomenon of tissue regeneration has been a dream in the imagination of human beings since the beginning of history, which can be observed in the mythology of many ancient civilizations. The idea of regenerating lost limbs and other parts of the body particularly gained momentum after technological advances in biotechnology in the 1980s and 1990s. More recent studies using stem cells rekindled the desire to deepen our understanding of this phenomenon. Since freshwater planarians are composed of pluripotent stem cells called neoblasts, and they are a good animal model for molecular studies, I believe that the key to understanding how to control tissue regeneration lies within the planarians.

What changes do you think could improve the professional lives of early-career scientists?

Greater, wider and more simple access to funding sources for students and early-career scientists, and if possible, government aid for institutes to cover publishing or additional research costs. In Brazil, we are currently experiencing a political and financial crisis that has spread through several sectors, meaning that many governmental research programs have been canceled or were drastically reduced. Brazilian researchers and scientists are struggling to fight the crisis and produce good scientific research with almost no means or financial incentives. I believe that science is one of the ways to rebuild the country, and this should begin with helping students access research through scholarships that give them the means to attend the laboratory of their choice daily.

What's next for you?

I really love to work with planarians - I have formed a relationship with them, they are lovely animals. I intend to start my doctorate as soon as I get access to a PhD fellowship, making use of methodologies such as spectroscopy and photobiomodulation in my project. In the meantime, I will continue working in several LAPLA studies as an invited researcher. What always motivates me to go to the laboratory is the challenge of working in scientific research.

Reference

Oliveira, M. S., Lopes, K. A. R., Leite, P. M. S. C. M., Morais, F. V. and Campos Velho, N. M. R. (2018). Physiological evaluation of the behavior and epidermis of freshwater planarians (*Girardia tigrina* and *Girardia* sp.) exposed to stressors. *Biol. Open* **7**: bio029595.
